# Use of Proton Pump Inhibitors and the Risk of Hospitalization for Infectious Gastroenteritis

**DOI:** 10.1371/journal.pone.0168618

**Published:** 2016-12-20

**Authors:** Yingxi Chen, Bette Liu, Kathryn Glass, Wei Du, Emily Banks, Martyn Kirk

**Affiliations:** 1 The Australian National University, Canberra, ACT, Australia; 2 University of New South Wales, Sydney, NSW, Australia; 3 Sax Institute, Sydney, NSW, Australia; University Hospital Llandough, UNITED KINGDOM

## Abstract

**Introduction:**

To quantify the association between PPI use, type and dose and infectious gastroenteritis hospitalization in a population-based cohort of middle-aged and older adults.

**Methods:**

Prospective study of 38,019 concession card holders followed up over 6 years in the Sax Institute’s 45 and Up Study. Data from the baseline questionnaire were linked to prescription medication, hospitalization, notifiable disease, cancer registry and death datasets from 2006–2012. Associations between PPI use and gastroenteritis hospitalization were examined using Cox regressions with age as the underlying time variable.

**Results:**

Among 38,019 participants, the median age was 69.7 years, and 57.3% were women. Compared to non-users, current PPI users were more likely to be older, and have a higher BMI. During follow-up there were 1,982 incident gastroenteritis hospitalizations (crude rate: 12.9 per 1000 person-years, 95% CI: 12.3–13.5). PPI use was significantly associated with infectious gastroenteritis hospitalization (aHR 1.4, 95% CI: 1.2–1.5). Among current users, a dose-response relationship was observed between the average daily dose (DDD) dispensed per day and infectious gastroenteritis hospitalization (*P*_trend_<0.001). We also observed higher rates of infectious gastroenteritis hospitalization and greater PPI use among participants with a history of chronic bowel problems (aHR 2.2, 95% CI: 1.9–2.5). There was no difference in risk by type of PPI. Recent use of H_2_ receptors was not associated with gastroenteritis hospitalization.

**Conclusion:**

PPI use is associated with an increased risk of infectious gastroenteritis hospitalization. Clinicians should be aware of this risk when considering PPI therapy.

## Introduction

Proton pump inhibitors (PPIs), introduced in 1989, are the most potent gastric acid suppressants available [1]. They are widely used by both gastroenterologists and primary care physicians in the effective treatment of acid-related disorders. PPIs are one of the most commonly prescribed medications worldwide [2], although it has been suggested that 25–70% of patients taking PPIs lack appropriate indications [2]. In Australia, there are five PPIs listed on the Pharmaceutical Benefits Scheme (PBS)—a national government system that subsidizes the cost of medicines, most of which are dispensed by pharmacists. Omeprazole and lansoprazole were first introduced onto the PBS in 1994, followed by pantoprazole in 1995, rabeprazole in 2001 and esomeprazole in 2002. Since their introduction, PPI use in Australia has grown dramatically [3]. In the 2013–14 financial year, physicians issued over 19 million prescriptions for PPIs with the most commonly prescribed type of PPI costing the PBS over $200 million [4].

Many patients take PPIs on a continuous or long-term basis [5]. Although this class of drug is considered safe and has been approved for long-term use [6], concerns have been raised regarding associated adverse effects [7]. Studies have reported that PPIs are associated with serious adverse events, including kidney diseases, hip fracture, community-acquired pneumonia, and *Clostridium difficile* infection [8–11]. PPIs irreversibly inactivate the gastric H^+^/K^+^-ATPase pump and cause a profound inhibition of gastric acid secretion [12, 13]. Significant hypochlorhydria, particularly among the elderly population who may have decreased clearance of PPIs, could result in bacterial overgrowth [14] and potentially increase susceptibility to infection. PPI use has also been shown to reduce gut commensal load and microbial density [15].

Although PPIs can potentially impair gastrointestinal host defenses, the association between PPI use and enteric infections has only recently been explored systematically [16, 17]. Observational studies have found increased risks of *Campylobacter*, *Salmonella* and *C*. *difficile* infection [9, 18]. However, the effects of different types and doses of PPIs remain unknown. Additionally, to our knowledge, no population-based studies have evaluated the effect of different PPI dosage and infectious gastroenteritis hospitalization among patients with chronic bowel problems. Given that older adults constitute the majority of PPI users [3, 19], the aim of this study was to investigate the association between PPI use and hospitalization for infectious gastroenteritis, considering both dose and type of PPIs, in a large prospective study of adults aged 45 years and older with and without a history of chronic bowel problems.

## Methods

### Data sources and study population

The Sax Institute’s 45 and Up Study is an Australian cohort of 267,153 men and women aged 45 years and over from New South Wales (NSW), the most populous state in Australia. The 45 and Up Study cohort were randomly selected from the Medicare Australia (now the Department of Human Services) enrolment database. Baseline questionnaires were distributed from 1 January 2006 to 31 December 2008. Participants joined the study by completing the baseline questionnaire and giving consent for follow-up through repeated data collection and linkage of their data to multiple population health databases. Baseline questionnaire data include information on socio-demographics, general health and behavior. The study is described in detail elsewhere [20], and questionnaires can be accessed at http://www.45andup.org.au.

For this report we linked individual participant baseline data to prescription medication, hospitalization, notifiable disease, cancer registrations and death datasets. Specifically, the 45 and Up Study baseline questionnaire data were linked to medication data from the PBS records to obtain medication use at baseline and during follow-up. Questionnaire data were linked to hospitalization data from the NSW Admitted Patient Data Collection (APDC) to identify cases of infectious gastroenteritis and to capture participants with previous hospitalizations. In order to identify cases of *Salmonella* infection, which is a notifiable disease in NSW, baseline data were linked to the Notifiable Conditions Information Management System (NCIMS). Data were then linked to death data to ascertain fact and date of death for censoring purposes. Baseline data were also retrospectively linked to cancer registry data from the NSW Central Cancer Registry (CCR) to identify participants who had a cancer diagnosis before recruitment. The NSW Centre for Health Record Linkage performed the data linkage independent of the study investigators and report false positive and false negative linkages of <0.5% and <0.1%, respectively [21].

The PBS dataset is an administrative dataset documenting information about subsided dispensed prescription drugs including PPIs for the Australian population [22]. For medicines listed on the PBS, consumers contribute a copayment towards the cost, and the Australian Government pays the remainder. People with a concession card pay a smaller copayment (AUD 6 in 2014) than the general population. Concession card holders are people with a Pensioner Concession Card, a Commonwealth Seniors Health Card or a Health Care Card. The PBS captured all medicines dispensed to concession card holders in the time period covered by these analyses.

The NSW APDC dataset is a complete census of all hospital admissions in NSW. The principal diagnosis for each admission, and up to 54 additional diagnoses contributing to the admission were coded using the International Classification of Diseases, 10^th^ revision, Australian Modification (ICD-10-AM) [23]. The NCIMS database contains a record of *Salmonella* infections in NSW, including the estimated onset date and the type of laboratory specimen used for confirmation. The NSW CCR is a population-based registry that records all new diagnoses of cancer in NSW residents and all deaths from cancer.

### Measurements

#### Case definition

The primary outcome of interest was hospitalization with infectious gastroenteritis, which was defined as a participant with an index linked hospitalization record where the principal or a secondary diagnosis was coded with an ICD-10-AM code for intestinal infectious diseases (A00-A09) following study recruitment.

Secondary outcomes included *Salmonella*-, *Campylobacter*- and *C*. *difficile* infection. A case of *Salmonella* infection was defined as a participant who had a linked notification record of non-typhoidal *Salmonella* infection during follow up. A case of *Campylobacter*-, or *C*. *difficile* infection was defined as a participant who had a linked hospitalization record with diagnosis of *Campylobacter* enteritis (ICD-10-AM code A04.5), or *C*. *difficile* colitis (A04.7) during follow up, respectively.

#### Definition of PPI use

PPI use was identified using linked records on dispensing from the PBS dataset with Anatomical Therapeutic Chemical (ATC) classification codes beginning with A02BC, proton pump inhibitors (World Health Organization Collaborating Centre for Drug Statistics Methodology, 2013) [24]. Study participants were categorized as current PPI users, former users and non-users. Current users were defined as those who had at least one PPI dispensing record within the 3 months prior to recruitment. Former users were defined as participants who had at least one PPI dispensing record in a period of 3–12 months prior to recruitment. Non-users were defined as participants who were not dispensed any PPIs over the period prior to recruitment that we had PBS records for, or who had a PPI dispensed ≥12 months prior to recruitment.

Current users were further categorized by type of PPI and dose. Types of PPIs used included omeprazole (ATC codes: A02BC01), pantoprazole (A02BC02), lansoprazole (A02BC03), rabeprazole (A02BC04), esomeprazole (A02BC05) or more than one type. Dose was described as the average number of dispensed DDD per day during the 3 months prior to recruitment [25]. DDD is a World Health Organization classification system which is defined as ‘*the assumed average maintenance dose per day for a drug used for its main indication in adults’* [26]. To obtain the average number of dispensed DDD per day, we firstly calculated the total number of dispensed DDD for each PPI, which was calculated as the strength (mg) of the dispensed PPI multiplied by the pack size and the number of dispensed packs, and then divided by the DDD of that PPI. This dispensed DDD was then summed for each participant and divided by the duration of use (3 months) to obtain the average dispensed DDD per day during the 3 months prior to the recruitment.

#### Definition of covariates

Socio-demographic factors and health status characteristics obtained from the baseline questionnaire included: age (grouped as 45–54, 55–64, 65–74 or ≥75 years), sex, body mass index (BMI: <18.5, 18.5–24.9, 25–29.9 or ≥30 kg/m^2^), self-rated health (excellent, good, fair or poor), smoking (current, past or never) and alcohol intake (none, 1–2 alcohol drinks per day or >2 alcohol drinks per day). Region of residence was obtained from Medicare Australia using address at time of recruitment, grouped as cities, inner regional or outer regional/remote based on the Accessibility/Remoteness Index of Australia [27].

History of cancer diagnosis, excluding non-melanoma skin cancer, in the 5 years prior to recruitment (yes, no) was ascertained by linkage to the CCR. History of chronic bowel problems (yes, no), was ascertained by linkage to an APDC record with an ICD-10-AM diagnosis code of K50 to K52 (non-infective enteritis and colitis) and K58 (irritable bowel syndrome) in any of the 55 diagnostic fields in the 6 years prior to recruitment. Recent H_2_ receptor antagonist and antibiotic use were defined based on the PBS dispensing records (ATC codes: A02BA01, A02BA02, A02BA03, A02BA04 for H_2_ receptors and J01 for antibiotics) in the 3 months before recruitment.

### Statistical methods

In this study, complete records of dispensed PPIs were only available for people with a valid healthcare concession-card [25]. Therefore, analyses were restricted to 45 and Up Study participants who were concession-card holders. Additionally, participants were excluded from the analyses if they had missing data on date of entry into the study, or missing PBS data on dispensing. Follow-up was calculated from the date of recruitment to the index date of admission for infectious gastroenteritis, death, or the last date for which database records were available (30 June 2012), whichever came first. Rates of infectious gastroenteritis hospitalizations since baseline and 95% confidence intervals (CIs) were calculated for PPI current users, former users and non-users at baseline.

Characteristics of PPI current users, former users and non-users were firstly compared using chi-squared tests. For the main analysis to examine the risk of PPI use and infectious gastroenteritis, Kaplan-Meier analysis with the log-rank test was first used to determine the probability of hospitalization with infectious gastroenteritis for current users, former users and non-users. Cox proportional hazards regression with age as the underlying time variable was then used to estimate the hazard ratios (HRs) and 95% confidence intervals (CIs). Regression models were initially adjusted only for age (as this was the underlying time variable) and sex, and then further adjusted for region of residence, self-rated health, BMI, cancer in previous 5 years, history of chronic bowel problems, recent H_2_ receptor antagonist use, and recent antibiotic use. Finally, smoking and alcohol intake were also added to the model.

In current users, the risk of infectious gastroenteritis hospitalization was further evaluated according to type and dose of PPIs. To examine the potential impact of chronic bowel problems on the association between PPI use and infectious gastroenteritis, models were then stratified by history of chronic bowel problems. Similar analyses were performed for the secondary outcomes of *Salmonella*-, *Campylobacter*-, and *C*. *difficile* infection, respectively.

The proportionality assumption of the Cox regression models were verified by plotting the Schoenfeld residuals against the time variable in each model, with the time-dependent form of the model used where covariates displayed non-proportionality of hazards. No violations were detected for PPI use. Significant violation was observed for recent antibiotic use, and this covariate was included as a time-dependent form in the models.

Sensitivity analysis was conducted by restricting cases to only those with a principal hospital diagnosis of infectious gastroenteritis. To examine the effects of changes in PPI use over time a second sensitivity analysis was conducted by restricting the study population to participants who remained in the same PPI use category during follow-up. We then conducted a third sensitivity analysis using a time-dependent Cox model with time-varying PPI ever-use. All analyses were carried out using STATA 12.1.

### Ethics approval

The conduct of the 45 and Up Study was approved by the University of New South Wales Human Research Ethics Committee. Ethics approval for this study was obtained from the NSW Population and Health Services Research Ethics Committee, and the Australian National University Human Research Ethics Committee. All participants provided written informed consent.

## Results

After restricting participants to those with concessional-only PBS records during the study period (n = 38,074), and excluding those who had missing data on date of entry into the study (n = 10), or missing PBS data on dispensing (n = 45), there were 38,019 participants, who were followed from baseline for a median of 3.9 years, yielding a total of 153,997 person-years of follow-up. The median age of study participants at recruitment was 69.7 years (interquartile range: 63.3–77.4), and 57.3% were women.

Table 1 summarizes the characteristics of the study population. Overall, 52.1% (n = 19,787) of participants had been dispensed at least one PPI in the 3 months prior to recruitment (categorized as PPI current users), 38.8% (n = 14,762) were defined as non-users, of which 18.8% (n = 2,771) had a record of PPI use ≥12 months before recruitment. PPI current users were more likely to be older and have a higher BMI compared to non-users. Participants taking H_2_ receptor antagonists had similar characteristics to participants taking PPIs (Table 1).

**Table 1:** Baseline characteristics of the study population, the 45 and Up Study, according to use of Proton Pump Inhibitors (PPI) and H_2_ receptor antagonists.

Characteristics	PPI non-users (n = 14,762)(%)	PPI former users (n = 3,470) (%)	PPI current users (n = 19,787) (%)	H_2_ receptor users (n = 1,951) (%)	*P*-value*
**Age (years)**					*P* < .001
45–54	1,564 (10.6)	366 (10.6)	1,376 (6.9)	152 (7.8)	
55–64	3,440 (23.3)	728 (21.0)	4,102 (20.8)	384 (19.6)	
65–74	5,503 (37.3)	1,354 (39.0)	7,517 (38.0)	729 (37.4)	
≥75	4,255 (28.8)	1,022 (29.4)	6,792 (34.3)	686 (35.2)	
**Female sex**	8,336 (56.5)	2,043 (58.9)	11,408 (57.6)	1,136 (58.2)	*P =* .3
**Region of residence**					*P* = .03
Cities	5,898 (39.9)	1,488 (42.8)	7,898 (39.9)	881 (45.1)	
Inner regional	5,467 (37.1)	1,245 (35.9)	7,568 (38.3)	646 (33.1)	
Outer regional/remote	3,397 (23.0)	737 (21.3)	4,321 (21.8)	424 (21.7)	
**History of chronic bowel problems**	692 (4.7)	249 (7.2)	1,752 (8.9)	158 (8.1)	*P* < .001
**Self-rated health**					*P* < .001
Excellent	4,919 (33.3)	1,053 (30.4)	4,670 (23.6)	422 (21.6)	
Good	5,484 (37.2)	1,258 (36.3)	7,569 (38.3)	698 (35.7)	
Fair	2,961 (20.1)	765 (22.1)	5,108 (25.8)	531 (27.2)	
Poor	656 (4.4)	192 (5.5)	1,436 (7.3)	177 (9.1)	
**Cancer in previous 5 years**	1,184 (8.0)	299 (8.6)	1,760 (8.9)	155 (7.9)	*P =* .03
**BMI (kg/m**^**2**^**)**					*P* < .001
<18.5	233 (1.7)	63 (1.9)	288 (1.6)	25 (1.4)	
18.5–24.9	4,682 (34.5)	1,127 (35.5)	5,252 (28.9)	601 (33.5)	
25–29.9	5,125 (37.7)	1,164 (36.7)	6,999 (38.6)	633 (35.3)	
>30	3,550 (26.1)	818 (25.9)	5,576 (30.8)	535 (29.8)	
**Smoking**					*P* < .001
Never	7,610 (51.9)	1,829 (53.0)	10,195 (51.8)	1,021 (52.9)	
Current	1,457 (9.9)	311 (9.1)	1,375 (7.0)	158 (8.2)	
Past	5,599 (38.2)	1,308 (37.9)	8,079 (41.2)	751 (38.9)	
**Alcohol intake**					*P* < .001
None	6,349 (43.0)	1,534 (44.2)	9,053 (45.7)	955 (48.9)	
≤2 units/day	6,211 (42.1)	1,431 (41.2)	7,951 (40.2)	718 (36.8)	
> 2 units/day	1,644 (11.1)	354 (10.2)	2,104 (10.6)	186 (9.5)	

*P*-value*: Chi-squared test for PPI category. Missing: self-rated health = 1,948 (5.1%); BMI = 3,142 (8.2%); smoking = 256 (0.6%); alcohol intake = 1,388 (3.6%)

Among users, esomeprazole was the most frequently dispensed PPI (n = 5,950; 30.1%), followed by omeprazole (n = 4,983; 25.2%) and pantoprazole (n = 4,235; 21.4%). Most users had used only one type of PPI (n = 19,096; 96.1%).

**Fig 1:**
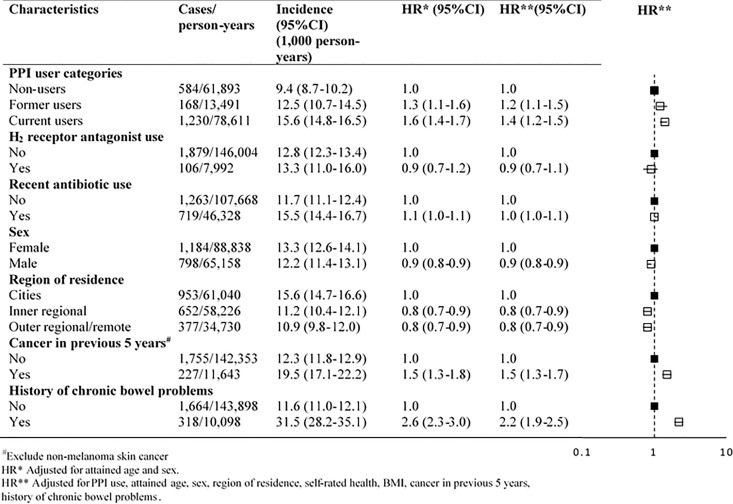
Crude incidence and hazard ratios of participants admitted to hospital with infectious gastroenteritis according to Proton Pump Inhibitor (PPI) user categories and other characteristics.

There were 1,982 cases of incident infectious gastroenteritis hospitalization during follow-up. The crude incidence of gastroenteritis hospitalization in the cohort was 12.9 per 1,000 person years (95% CI, 12.3–13.5). Compared to non-users, the adjusted relative risk of hospitalization was significantly higher in current PPI users (aHR 1.4, 95% CI: 1.2–1.5) and former users (aHR 1.2, 95% CI: 1.1–1.5) (Fig 1). Recent use of prescribed H_2_ receptors was not associated with hospitalization for infectious gastroenteritis (aHR 0.9, 95% CI: 0.7–1.1). Participants with a history of cancer or chronic bowel problems were more likely to be hospitalized with infectious gastroenteritis (aHR 1.5, 95% CI: 1.3–1.7; and 2.2, 95% CI: 1.9–2.5, respectively).

Among current users, a dose-response relationship was observed between the average number of DDD dispensed per day and risk of infectious gastroenteritis hospitalization (*P*_trend_<0.001), with a 60% increase in risk among those dispensed >1 DDD/day versus non-use (aHR 1.6, 95% CI: 1.3–1.8). The risk did not differ significantly by PPI type (Table 2). The dose response effect was consistent when analyses were restricted to participants with a history of chronic bowel problems; compared to non-users, aHRs of infectious gastroenteritis hospitalization were 1.2 (95% CI: 0.8–1.9) in participants with a dose ≤0.5DDD/day, 1.7 (95% CI: 1.2–2.2) with a dose of 0.5-1DDD/day, and 2.0 (95% CI: 1.4–2.8) with a dose >1DDD/day (*P*_trend_<0.001) (Table 2).

**Table 2:** Hazard ratios of participants hospitalized with infectious gastroenteritis among current Proton Pump Inhibitor (PPI) users compared to non-users according to dose and type of PPI.

Characteristics	HR* (95%CI)	*P*_trend_	Participants with chronic bowel problems			Participants without chronic bowel problems		
			Rate^#^	HR** (95%CI)	*P*_trend_	Rate^#^	HR** (95%CI)	*P*_trend_
**Average daily dose (DDD)**		<0.001			<0.001			<0.001
Non-users	1.0		22.1	1.0		9.4	1.0	
≤0.5	1.1 (0.9–1.3)		25.2	1.2 (0.8–1.9)		10.8	1.1 (0.9–1.3)	
0.5–1	1.4 (1.3–1.6)		37.5	1.7 (1.2–2.2)		14.3	1.4 (1.3–1.6)	
>1	1.6 (1.3–1.8)		45.1	2.0 (1.4–2.8)		15.9	1.5 (1.3–1.8)	
**Type of PPI**		0.2			0.3			0.4
Omeprazole	1.0		36.7	1.0		14.9	1.0	
Pantoprazole	0.9 (0.8–1.1)		38.8	1.1 (0.7–1.6)		12.7	0.9 (0.7–1.1)	
Lansoprazole	0.9 (0.6–1.2)		21.6	0.7 (0.3–1.7)		12.7	0.9 (0.6–1.2)	
Rabeprazole	1.1 (0.9–1.3)		44.8	1.3 (0.8–2.0)		14.4	1.1 (0.8–1.3)	
Esomeprazole	0.9 (0.8–1.1)		33.1	0.9 (0.6–1.4)		13.0	1.0 (0.8–1.1)	

Rate^#^ /1,000 person-years. HR* Adjusted for age, sex, region of residence, self-rated health, BMI, cancer in previous 5 years, history of chronic bowel problems, H_2_ receptor antagonist use, recent antibiotic use, smoking and alcohol consumption. HR** Adjusted for age, sex, region of residence, self-rated health, BMI, cancer in previous 5 years, recent H_2_ receptor antagonist use, recent antibiotic use, smoking and alcohol consumption.

The broad relationships between PPI use and the risk of specific types of infectious gastroenteritis—*C*. *difficile*, *Salmonella* and *Campylobacter* infection—did not differ materially from that observed for infectious gastroenteritis hospitalization overall. Risks were significantly elevated for *C*. *difficile* infection among PPI current users (aHR: 1.5, 95% CI: 1.1–2.1). Compared to non-users, participants dispensed >1 DDD/day were 120% more likely to have *C*. *difficile* infection (aHR: 2.2, 95% CI: 1.4–3.4), and 100% more likely to have *Salmonella* infection (aHR: 2.0, 95% CI: 1.1–3.8). While not statistically significant in all cases, there was a pattern of increased risk of these outcomes with increasing PPI dose, and this pattern was not generally observed for H_2_ receptor antagonists (Table 3).

**Table 3:** Proton Pump Inhibitor (PPI) use and the risk of *Salmonella*-, *Campylobacter*-, and *Clostridium difficile*-infection.

Characteristics	*Salmonella* infection (n = 84)	*Campylobacter* infection (n = 71)	*C*. *difficile* infection (n = 147)
	No. of events	No. of events	No. of events
PPI Non-users	30	21	43
PPI former users	3	5	7
PPI current users	51	45	97
**Association between medication use and infections**	HR* (95%CI)	HR* (95%CI)	HR** (95%CI)
**PPI use**			
Non-users	1.0	1.0	1.0
Former users	0.5 (0.2–1.6)	1.1 (0.4–2.9)	0.7 (0.3–1.6)
Current users	1.2 (0.7–1.8)	1.6 (1.0–2.7)	1.5 (1.1–2.1)
**H**_**2**_ **receptor antagonist use**			
No	1.0	1.0	1.0
Yes	1.1 (0.3–3.3)	2.3 (0.9–5.9)	0.6 (0.2–1.7)
**Antibiotic use**			
No	1.0	1.0	1.0
Yes	1.4 (0.9–2.3)	1.0 (0.6–1.6)	1.1 (0.8–1.5)
**Average daily dose (DDD)**			
Non-users	1.0	1.0	1.0
Current users: ≤0.5	0.8 (0.3–1.9)	1.4 (0.6–3.1)	1.2 (0.6–2.1)
Current users: 0.5–1	1.3 (0.8–2.1)	1.8 (1.1–3.0)	1.3 (0.9–2.0)
Current users: >1	2.0 (1.1–3.8)	1.1 (0.4–2.7)	2.2 (1.4–3.4)

HR* Adjusted for age, sex, region of residence, self-rated health, recent H_2_ receptor antagonist use, recent antibiotic use, cancer in previous 5 years, history of chronic bowel problems, and alcohol consumption. BMI and smoking status were not included in the model due to missing values in certain categories. HR** Adjusted for age, sex, region of residence, self-rated health, BMI, recent H_2_ receptor antagonist use, recent antibiotic use, cancer in previous 5 years, history of chronic bowel problems, smoking and alcohol consumption.

### Sensitivity Analyses

The results remained similar when restricting cases to only those with a principal hospital diagnosis of infectious gastroenteritis; compared to non-users, aHRs were 1.7 (95% CI: 1.4–1.9) for current users and 1.5 (95% CI: 1.2–2.0) for former users. A significant dose-response relationship was also observed; compared to non-users, aHRs were 1.1 (95% CI: 0.9–1.4), 1.4 (95% CI: 1.2–1.7) and 2.1 (95% CI: 1.8–2.5) in participants with a dose ≤0.5, 0.5–1 and >1DDD/day, respectively (*P*_trend_<0.001). In the second sensitivity analysis with PPI use as a time-varying covariate, PPI use was also associated with infectious gastroenteritis hospitalization (aHR: 1.9, 95% CI: 1.6–2.1). Associations remained similar when further restricting the study population to participants who did not change PPI use category during follow-up. A similar dose-response relationship was retained in this analysis (*P*_trend_<0.001).

## Discussion

In this study, we found a significantly increased risk of infectious gastroenteritis hospitalization associated with PPI use, and a significant dose-response relationship among current users. This risk was specific to PPI users, as use of H_2_ receptor antagonists, which are used for the same indication as PPIs, was not associated with hospitalization due to infectious gastroenteritis. This study confirms that the risk of infectious gastroenteritis hospitalization is elevated in people who have used PPIs, and also provides new and reliable information about the effects of different types of PPIs and dosages.

We found that former and current PPI users had significantly increased risks of infectious gastroenteritis hospitalization compared to those never using or using PPIs ≥12 months prior to baseline. Previous studies have reported current PPI therapy as a significant risk factor for bacterial gastroenteritis [18, 28]. Howell *et al* reported increasing rates of nosocomial *C*. *difficile* infection with increasing level of PPI therapy [29]. In this study, we observed a significant dose-response relationship between PPI exposure and all-cause infectious gastroenteritis hospitalization, which has not been demonstrated previously. This dose-response relationship, and the fact that it is specific to PPIs and was not seen in users of H_2_ receptor antagonists, supports a causal association. In this study, we found a small elevation in risk of gastroenteritis hospitalization among former users, which has not been investigated previously; it may be due to long-term effects of PPI use, but requires confirmation.

The reason for the association between PPI use and infectious gastroenteritis is not known definitively, although colonization and proliferation of pathogens secondary to acid suppressive treatment is one potential explanation. Gastric acid plays an important role in preventing human gastrointestinal infections [30] and an acidic environment in the upper gastrointestinal tract constitutes one of the major non-specific defenses to protect against ingested microorganisms [31]. Acid suppression induced by PPIs also affects gastrointestinal motility and can indirectly alter gut microbiota [32]. In patients with functional bowel disorders, such as irritable bowel syndrome, such changes could be more pronounced [33, 34]. Our study found that PPI use resulted in elevated risk of infectious gastroenteritis hospitalization in people with and without a history of chronic bowel problems. We also observed higher rates of infectious gastroenteritis hospitalization and greater PPI use among participants with a history of chronic bowel problems, indicating greater absolute risks of PPI-attributable hospitalization in this group. This suggests that the necessity for PPI use may need to be evaluated more carefully in this group of patients.

Previous studies have reported associations between PPI use and enteric infections, such as *Campylobacter*, *Salmonella* [18] and *C*. *difficile* infection [35]. We found a broad association between PPI use and *Campylobacter-*, *Salmonella-* and *C*. *difficile* infection. While the association was not statistically significant in all cases, which could be due to smaller number of events, there was a pattern of increased risk of infections in PPI users and potential dose-response relationships. In addition, our prior work using the full 45 and Up Study dataset showed a significant risk of *Salmonella* infection among people who self-reported PPI use at baseline (aHR 1.87, 95%CI 1.43–2.40) [36]. Our findings regarding *C*. *difficile* infection were consistent with published data. A recent systematic review of 39 studies showed PPI users at higher risk of *C*. *difficile* infection compared to non-users (odds ratio: 1.74, 95%CI 1.47–2.85) [9]. Based on latest evidence, the FDA have published safety alerts warning of the association between *C*. *difficile* diarrhea and PPIs [37]. In Europe, PPI use for more than 8 weeks at the maximal dose without clear indication has been listed on the European list (EU [7)-PIM list) of potentially inappropriate medications for older people due to the association between PPI use and *C*. *difficile* infection [38].

To ensure that the study focused on the likely causal effect of PPI use on infectious gastroenteritis hospitalization, comorbidity status was controlled through adjustments of cancer history, general health and BMI. Participants with digestive disorders may be more likely to be prescribed acid suppressive medications. These patients also may be more likely to experience infectious gastroenteritis and be hospitalized. It was not possible to account for all possible digestive disorders, although we identified participants with chronic bowel problems at baseline and adjusted for them in the regression models. We also stratified results by the status of chronic bowel problems. To examine the effect of confounding by indication of acid suppressive therapy, we considered recent H_2_ receptor antagonist use in the analysis. Similar to PPIs, H_2_ antagonists are a class of acid suppressants used to treat acid-related disorders such as peptic ulcers. We did not observe any increased risk of infection among H_2_ receptor antagonist users, indicating that confounding by indication was unlikely to be a major source of bias in this study.

The large number of cases in the study enhanced the precision of the estimates, and allowed adequate assessment of the effects of potential confounders. However, we were only able to classify based on medication usage from dispensing data rather than directly observed therapy, meaning we were unable to confirm actual PPI use in this study. Misclassification relating to non-use among those with records of having been dispensed PPIs would tend to lead to an overestimation of the potential risk of PPIs. However, this bias would be unlikely to affect the assessment of dose, as it is less likely that patients with multiple dispensing records of PPIs did not take the medication. In addition, a recent systematic review suggested that the majority of patients with GERD are relatively adherent to PPIs, and adherence increases with severe symptoms [39]. Secondly, as with most observational studies, residual confounding by unmeasured factors is a potential concern. In this study, we controlled for several important confounders, although we were unable to assess other factors, such as use of over-the-counter antacids. During the study period, low-dose PPIs were available from pharmacies without a prescription in Australia, which could lead to misclassification of PPI exposure. Thirdly, inpatient hospitalization data can be subject to misclassification. However, sensitivity analysis restricting cases only to principal diagnosis of infectious gastroenteritis showed similar results to the main findings. Finally, the study population was restricted to concession-card-holders. Therefore, participants were likely to be older, with lower socio-economic status, when compared to the broader cohort, although risk factor estimates are considered broadly generalizable from within-cohort comparisons [40].

In summary, PPI use is associated with an increased risk of infectious gastroenteritis hospitalization in the 45 and Up Study participants, with higher risks with increasing doses. Given the widespread use of PPIs, particularly among the elderly, clinicians should be aware of this risk when considering PPI therapy, and use the lowest effective dose for patients with appropriate indications. For patients with chronic bowel problems, it may be worth considering an alternative dosage or switching to H_2_ receptor antagonists.
